# CRH Affects the Phenotypic Expression of Sepsis-Associated Virulence Factors by *Streptococcus pneumoniae* Serotype 1 *In vitro*

**DOI:** 10.3389/fcimb.2017.00263

**Published:** 2017-06-22

**Authors:** Colette G. Ngo Ndjom, Lindsay V. Kantor, Harlan P. Jones

**Affiliations:** ^1^Department of Molecular and Medical Genetics, University of North Texas Health Science CenterFort Worth, TX, United States; ^2^Graduate School of Biomedical Sciences, University of North Texas Health Science CenterFort Worth, TX, United States

**Keywords:** *Streptococcus pneumoniae*, serotypes, corticotropin releasing hormone, sepsis virulence, phenotype

## Abstract

Sepsis is a life-threatening health condition caused by infectious pathogens of the respiratory tract, and accounts for 28–50% of annual deaths in the US alone. Current treatment regimen advocates the use of corticosteroids as adjunct treatment with antibiotics, for their broad inhibitory effect on the activity and production of pro-inflammatory mediators. However, despite their use, corticosteroids have not proven to be able to reverse the death incidence among septic patients. We have previously demonstrated the potential for neuroendocrine factors to directly influence *Streptococcus pneumoniae* virulence, which may in turn mediate disease outcome leading to sepsis and septic shock. The current study investigated the role of Corticotropin-releasing hormone (CRH) in mediating key markers of pneumococcal virulence as important phenotypic determinants of sepsis and septic shock risks. *In vitro* cultures of serotype 1 pneumococcal strain with CRH promoted growth rate, increased capsule thickness and penicillin resistance, as well as induced pneumolysin gene expression. These results thus provide significant insights of CRH–pathogen interactions useful in understanding the underlying mechanisms of neuroendocrine factor's role in the onset of community acquired pneumonias (CAP), sepsis and septic shock.

## Introduction

Sepsis is a life-threatening health condition caused by infectious pathogens that may be acquired through communal contact and in hospital settings (Page et al., 2015). Disease pathogenesis is defined by breech of localized respiratory infection which precipitates into an uncontrolled systemic activation of multiple cellular immune and inflammatory pathways (Russell, 2006; Boomer et al., 2011). In cases of severe disease, conditions can manifest, involving hypotension and organ dysfunction, at which point it is referred to as *septic shock*. In the United States alone, 28–50% of sepsis patients die annually (Hall et al., 2011), exceeding the number of total deaths from prostate cancer, breast cancer, and AIDS (Wood and Angus, 2004). Furthermore, intensive care unit (ICU) patients are at higher risk of developing or being treated for sepsis (Martin, 2012), costing an average of $1,878 *per diem* for state/local government hospitals and $2,289 *per diem* for non-profit hospitals (Rappleye, 2015).

Administration of corticosteroids is a common treatment of sepsis due to its broad inhibitory effect on the activity and production of pro-inflammatory mediators including cytokines, chemokines, eicosanoids, bradykinin, and activator protein-1 (Prigent et al., 2004). Yet, despite its ability to counteract overt inflammatory responses and restore cardiovascular homeostasis and organ function (Annane, 2011), the increase in the number of deaths raises questions regarding its clinical benefit. For example, analyses of randomized trials have suggested that administration of low-to-moderate doses of corticosteroids can improve survival (Annane et al., 2009; Minneci et al., 2009; Moran et al., 2010); whereas other meta-analyzes have demonstrated that corticosteroid regimens were not beneficial in sepsis and septic shock (Minneci et al., 2009; Sligl et al., 2009). Specifically, analysis of a 2005 clinical trial found that short course administration of corticosteroids did not reverse the mortality rates from sepsis, and in some instances increased the incidence of nosocomial infections, as well as of organ dysfunction (Beutz and Abraham, 2005). More recently, a meta-analysis of 35 randomized clinical trials evaluating steroids on sepsis patients did not yield a statistically significant beneficial effect of the use of corticosteroids in the treatment of sepsis (Volbeda et al., 2015). A deficiency in clear-cut evidence supporting or negating the benefits of corticosteroids in the treatment of sepsis highlights the complexity of this condition, and warrants a better understanding of the mechanisms of action.

The respiratory tract is the source of sepsis in 40–60% of patients (Nasa et al., 2012; Riyaz et al., 2014). *Streptococcus pneumoniae* (*S. pneumoniae)* is a common respiratory pathogen responsible for most community acquired pneumonias (CAP) cases worldwide (Lynch and Martinez, 2002). It is also a major cause of nosocomial associated sepsis mortality (Hifumi et al., 2016). Whereas restricted inflammatory immune responses of the respiratory tract are beneficial, excessive activation may elicit systemic manifestations of sepsis (Chen et al., 2011; Bosmann and Ward, 2013). Host protection against sepsis is influenced by endogenous glucocorticoid activity (e.g., cortisol release) controlled by the hypothalamic-pituitary-adrenal axis (HPA), that maintains homeostatic regulation of cellular immune and inflammatory responses (Wilson et al., 1998). However, a loss or deficiencies in HPA-mediated responses do occur (Annane, 2016), resulting in disruptions in normal adrenal axis functioning. This may cause hyper- or hypo-activation of the immune response against *S. pneumoniae*. Uncontrolled inflammatory responses have been shown to be critical determinants of sepsis and septic shock among patients diagnosed with community-acquired and nosocomial-associated *S. pneumoniae* (Sam et al., 2004; den Brinker et al., 2005).

The prognostic value of cortisol levels has been reported in sepsis and other critical illnesses (Annane et al., 2008) in correlation with the status of cellular immune and inflammatory mediators (Marik and Zaloga, 2002). In recent years, however, emphasis has shifted from determining the cross-talk between the neuroendocrine and immune systems to defining pathogens' direct response to neuroendocrine factors (Butts and Sternberg, 2008; ThyagaRajan and Priyanka, 2012; Procaccini et al., 2014). Corticotropin Releasing Hormone (CRH) is a 41-amino acid neuropeptide secreted from the HPA, and stimulates the release of adrenal cortisol in response to infection and other stressors (Nezi et al., 2015). CRH is expressed in peripheral tissues (e.g., lung) and by immune cell populations, and has been shown to impact inflammatory disease (Crofford et al., 1992; van Tol et al., 1996; Benou et al., 2005; Kokkotou et al., 2006; Kalantaridou et al., 2007). In support, we have previously demonstrated a role for peripheral CRH in manipulating immune and inflammatory responses against *S. pneumoniae* respiratory infection (Gonzales et al., 2008; Kim et al., 2011). Our findings demonstrated an association between CRH expression in the lungs of mice with severe pneumonia and increased lung and systemic disease (Gonzales et al., 2008). We also found that pre-exposing *S. pneumoniae* to CRH increased bacterial counts in the lung. Furthermore, direct exposure to CRH resulted in increased bacterial growth, and Pneumococcal-associated virulence factor A (pavA) gene expression (Ndjom and Jones, 2015). Collectively these results suggest a possible correlation between a bacterial pathogen's response to stress hormones (e.g., glucocorticoids) and the risk for developing sepsis and septic shock. In this study, we determined the role of CRH in mediating capsular phenotype and pneumolysin expression, as well as antibiotic sensitivity as key markers of pneumococcal virulence. The results from the current study provide significant insights of CRH–pathogen interactions useful in understanding the underlying mechanisms of neuroendocrine factor's role in disease pathogenesis.

## Materials and methods

### Bacterial strain and growth conditions

*Streptococcus pneumoniae* strain 6301, serotype 1 (ATCC, Manassas, VA, USA) was used in all experiments. Prior to use, *S. pneumoniae was* maintained in 30% glycerol frozen stock solutions and stored at −80°C. For inoculum preparation, *S. pneumoniae* was grown at 37°C, 5% CO_2_ either on Blood Agar plates (TSA w/5% Sheep Blood) (Life Technologies, Carlsbad, CA) or in Brain Heart Infusion broth (BHI) (Sigma Aldrich, St. Louis, MO).

#### Corticotropin-releasing hormone (CRH)

Human/Rat recombinant CRH (Sigma Aldrich, St. Louis, MO) was used in all experiments. CRH stock solutions were stored at −20°C in 20 μl aliquots until use.

### Pneumococcal growth curve

*Streptococcus pneumoniae* was grown overnight to achieve mid-log phase cultures on Blood Agar plates (Life Technologies, Carlsbad, CA). Twenty-four hours later, bacteria were collected and diluted in BHI until an optical density (O.D.) of 0.2 was achieved. A series of triplicate tubes were prepared containing 1,950 μl of BHI and 50 μl of *S. pneumoniae* in the presence or absence of either 4.0 × 10^−4^ or 8.0 × 10^−4^ mM/μl concentration of CRH. The tubes were incubated at 37°C, 5% CO_2_. Triplicate absorbance readings (OD_600_) of bacterial cultures were taken every 30 min. Absorbance readings were plotted against time to generate bacterial growth curves.

### Capsular staining of *S. pneumoniae*

*Streptococcus pneumoniae* was stained for capsular visualization as previously described by Hughes and Smith (2007). Briefly, 50 μl (1 × 10^5^ organisms) of bacterial suspension taken from frozen stock was exposed to CRH vs. control (broth only) and grown in 1 mL of BHI for 5 h, at 37°C and 5% CO_2_. Microscopy slides were prepared with 2–3 μl drops of Congo red solution (Carolina Biological, Burlington, NC) to which 6 μl of bacterial suspension were mixed. Slides were air dried before being flooded with Maneval solution (Carolina Biological, Burlington, NC) for 1 min. Stained slides were then gently rinsed off with deionized (DI) water, placed on absorbent paper and left to air dry. Slides were examined under oil immersion at 100X magnification. Capsular staining images were captured using Olympus AX70 Provis microscope (NY, USA). Capsular thickness was calculated as the difference between the total diameter of the bacterial cell from its outer membrane and the total distance in the diameter of the inner capsular membrane. Three independent measures were taken of three different bacterial cells per three individuals slides were averaged and expressed as the percent difference in the mean capsular thickness of organisms exposed to CRH compared to the mean capsular difference of control (untreated) organisms. Capsular diameters were determined using Adobe Photoshop CS6 software (Adobe, San Jose, CA).

### Quantitation of *S. pneumoniae*'s antibiotic sensitivity

#### Preparation of antibiotic serial dilutions

Two hundred microliters of a 10,000 U/mL stock penicillin/streptomycin (Thermo Fisher Scientific Inc., Waltham, MA) solution were added to the first well (Row A) of a 96-well microplate, in triplicates for both the CRH and non-CRH groups (total of six wells). One hindered microliter of Mueller-Hinton broth (Sigma Aldrich, St. Louis, MO) were added to all the remaining wells of each row. A serial dilution of the concentrated antibiotic cocktail was performed in Mueller-Hinton broth, resulting in 10 different antibiotic concentrations (2,000, 1,000, 500, 225, 125, 62.5, 31.25, 15.62, 7.81, and 3.9 U/well). Plates were then incubated at 35°C for 24 h for evaporation purposes before being used in the Minimum Inhibitory Concentration (MIC) assay.

#### MIC assay

*Streptococcus pneumoniae* was grown overnight to achieve mid-log phase cultures on Blood Agar plates (Life Technologies, Carlsbad, CA). Bacteria were collected and diluted in Mueller-Hinton broth (Sigma Aldrich, St. Louis, MO) until an optical density (O.D.) of 1 was achieved, representing a cell density of 5 × 10^8^ colony forming units (CFU)/mL. Twenty-five microliters of diluted bacteria were added onto the wells of a fresh 96-well (antibiotic free) microplate, representative of both CRH-treated and CRH-untreated groups. Twenty microliter of CRH (4.0 × 10^−4^ mM/μl) were added to each well of the CRH-treated group, followed by 200 μl of Mueller-Hinton broth and Defibrinated Sheep Blood (Sigma Aldrich, St. Louis, MO) mixture. In the CRH-untreated group, only the broth/blood mixture were added to the wells containing bacterial suspension. The control groups, free of antibiotic cocktail, also received bacterial suspension along with the broth/blood mixture (±CRH). The plate was covered and incubated for 2 h at 37°C and 5% CO_2_. Post-incubation, each well was transferred onto the dehydrated penicillin/streptomycin 96-well microplate. The plate was read using a Synergy 2 multi-modal microplate reader plate reader (BioTek, Winooski, VT) for time zero (T0) and incubated overnight at 37°C and 5% CO_2_. The plate was again read 24 h post-incubation (T24). All measurements and experiments were performed in triplicates.

### Pneumolysin (Ply) mRNA gene expression by quantitative real-time polymerase chain reaction (qRT-PCR)

#### Bacterial RNA extraction

*Streptococcus pneumoniae* exposed to CRH vs. control (broth only) was collected 5 h after incubation in separate 3 ml sterile conical tubes in 1 mL of sterile BHI broth. Bacterial suspensions were transferred into 1.5 mL Eppendorf centrifuge tubes and spun down at 14,000 g for 1 min. The pellets were then stored in 100 μl of RNA*later* solution for subsequent RNA extraction. RNA extraction from bacterial cells was performed using a RiboPure RNA Purification Kit (Life Technologies, Carlsbad, CA).

#### Reverse transcription and quantitative real-time RT-PCR

A starting Total RNA concentration of 1 μg per reaction and MLV (Molony murine leukemia virus) reverse transcriptase (Promega Corp., Madison, WI) were used to generate bacterial cDNA. Post-cDNA synthesis, real-time PCR was performed using SYBR green-based amplification techniques. Briefly, PCR was performed in 20 μl reaction volume using the StepOne system (Applied Biosystems Inc., Foster City, CA). The expression of the housekeeping gene 16 s rRNA was used as an internal control to normalize target gene expression between samples. ΔΔ^CT^ = Δ^CT^ (target gene) − Δ^CT^ (16 s rRNA) formula was used to calculate gene expression in bacterial cells of pneumolysin (Ply) gene. Data was calculated by subtracting ΔΔ^CT^ of control group from the CRH-treated group for each target gene. Data was expressed as the fold difference in Ply mRNA expression, by normalizing the expression of untreated Ply as 1. Selected targets and housekeeping gene primer sets; Ply, and 16 s rRNA were purchased from Life Technologies, Carlsbad, CA (Table 1).

**Table 1:** List of primer sequences and target gene.

	**Sequence**
**HOUSE-KEEPING GENE Rogers et al., 2007**	
16S rRNA-Forward (5′–3′)	TGAGTTAACCGTAAGGAGCCA
16S rRNA-Reverse (3′–5′)	TCACCCCAATCATCTATCCCA
**PLY-2 (PNEUMOCOCCAL PNEUMOLYSIN 2) Cope et al., 2011**	
Forward (5′–3′)	CTACCCGATGAGTTTGTTGTT
Reverse (3′–5′)	TCCAGGATAGAGGCGACT

#### Statistical analysis

Statistical analysis was performed using GraphPad Prism Version 6.0 (GraphPad Software, San Diego, USA). For multi-experimental group analysis, data were subjected to one-way and two-way ANOVA (analysis of variance) followed by *post-hoc* tests (Newman-Keuls and Bonfferoni) for group differences. All data are expressed as means ± standard error of mean (SEM). A *p* ≤ 0.05 was considered significant.

## Results

### Introducing CRH during log-phase promotes pneumococcal growth

**Figure 1:**
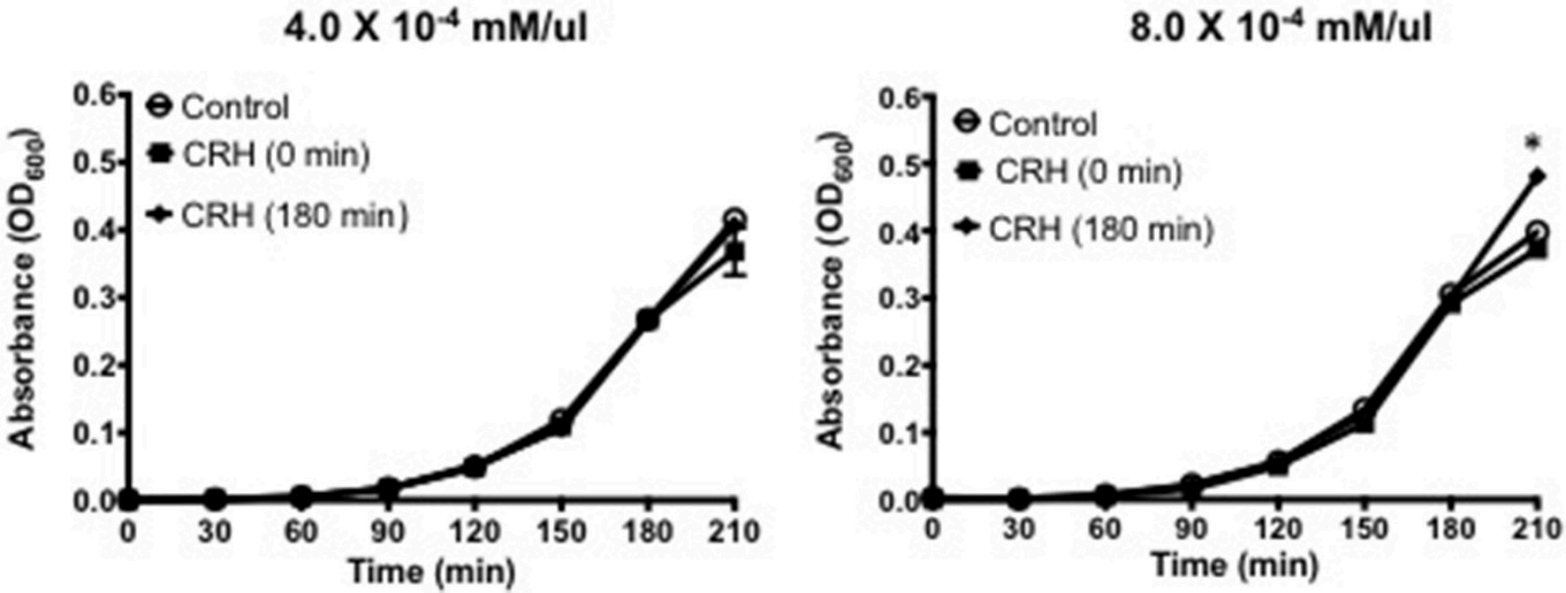
CRH promotes pneumococcal growth at log-phase. Growth curve analysis was performed on Serotype 1 pneumococcal strain in the presence or absence of CRH (4.0 × 10^−4^ and 8.0 × 10^−4^ mM/μl). Data represents mean (*n* = 3) ± standard deviation between CRH and control. Asterisks (^*^) indicate significant (*P* ≤ 0.05) differences between experimental and control groups.

The current study sought to determine whether growth could be influenced during *S. pneumoniae's* highest rate of growth (e.g., log-phase). As shown in Figure 1, introducing CRH (8 × 10^−4^ mM/μl) during log phase resulted in a significant increase in bacterial growth compared to exposure of CRH during lag phase growth. The growth curve was carried out over a 9 h period with now differences in growth observed beyond the 180-min point. Figure 1 shows the effect of CRH as observed up to 180 min. These findings demonstrate a dependency on the growth phase of *S. pneumoniae* to be influenced by CRH.

### CRH increases capsular formation

**Figure 2:**
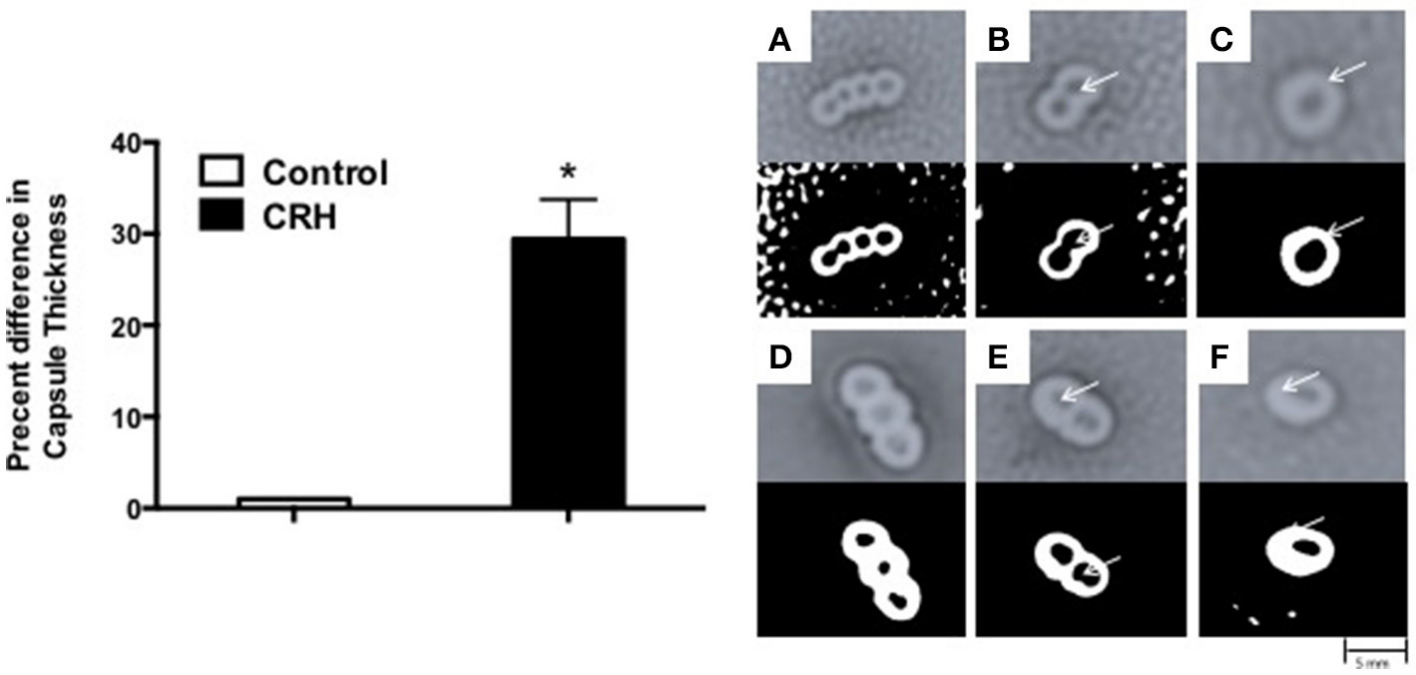
Capsular thickness is increased by CRH. Serotype 1 pneumococcal strain (10^8^ organisms) was grown in the presence or absence of CRH (4.0 × 10^−4^ mM/μl) for 5 h. Capsular thickness was determined by calculating the absolute difference between total diameter of outer and inner capsular membranes. Bars represent the mean (*n* = 9) ± standard deviation in the percent difference in capsular diameters of CRH-treated organisms compared to control. Asterisks (^*^) indicate significant (*P* ≤ 0.05) differences between experimental and control groups (Left). 100X magnified images of untreated **(A–C)** and CRH-treated **(D–F)** *S. pneumoniae* are shown in right panel. Lower panels represent corresponding high contrast color images highlighting capsule and diplonuclei denoted by arrows.

Capsule formation is a key virulence factor of *S. pneumoniae* (AlonsoDeVelasco et al., 1995) related to bacterial growth, attachment, biofilm integrity, and antibiotic resistance. It is also the source of many membrane bound and secreted proteolytic components owing to its pathogenesis (Wilson et al., 2002). The percent difference in capsule thickness was plotted for CRH treated groups against control (without CRH exposure). CRH exposure resulted in a significant increase in capsule thickness compared control (Figure 2). The increase in capsule formation in response to CRH was found to have a similar effect on three additional serotypes 3, 19A, and 23F (see Supplementary Figure 1).

### CRH increases antibiotic resistance

**Figure 3:**
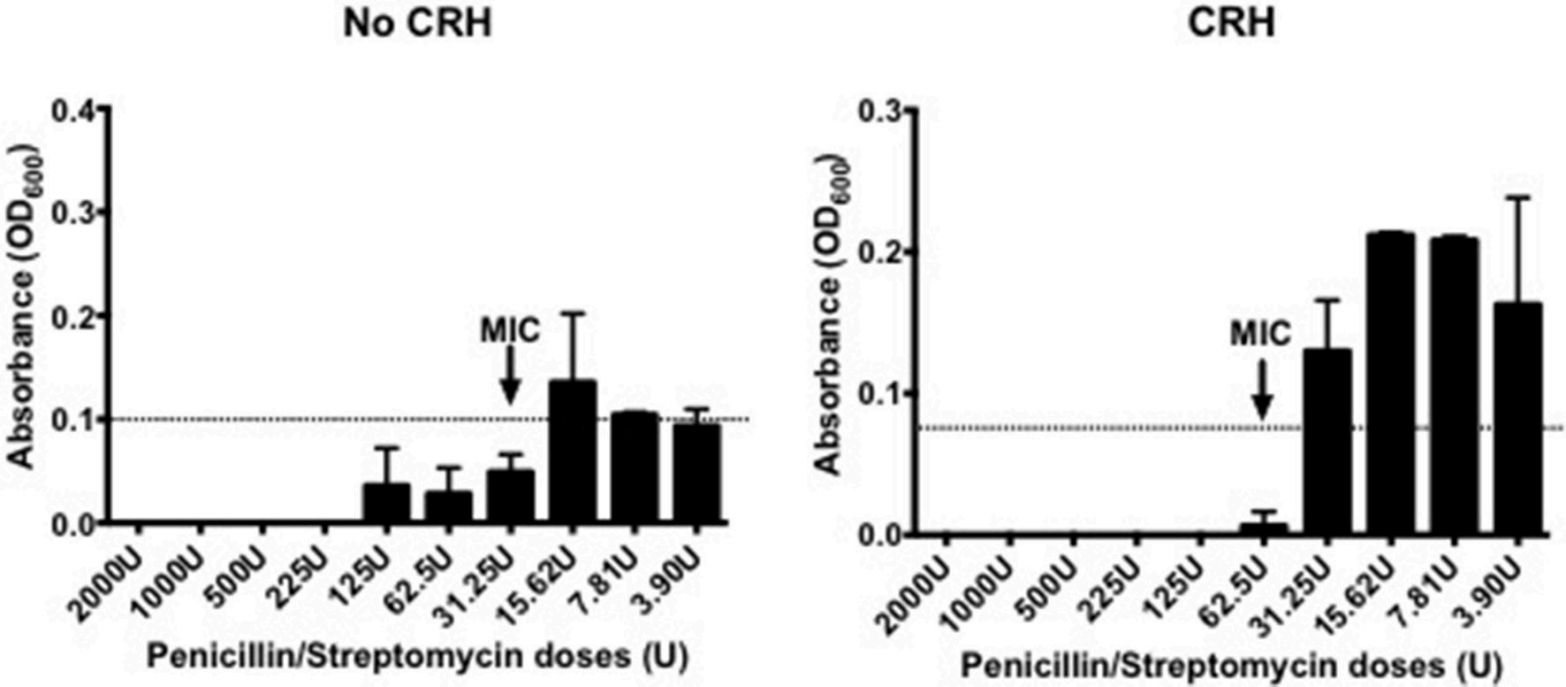
CRH increases antibiotic resistance. Serotype 1 pneumococcal strain were incubated for 2h in the presence or absence of CRH (4.0 × 10^−4^ mM/μl), and subsequently exposed overnight to various penicillin/streptomycin concentrations (2,000, 1,000, 500, 225, 125, 62.5, 31.25, 15.62, 7.81, and 3.9 U/well). Data represents one of three independent experiments performed in triplicate. Arrow indicates the minimal inhibitory concentration (MIC).

Capsule morphology influences the sensitivity to antibiotic targets. Given the effect by CRH on capsular thickness, studies were performed to determine whether CRH could have an impact on antibiotic resistance in *S. pneumoniae*. As such, CRH was introduced to bacterial cultures followed by incubation with different concentrations of Penicillin/Streptomycin (P/S) cocktail. CRH exposed *S. pneumoniae* showed a greater than three-fold resistance to P/S cocktail when compared to the untreated bacterial group (Figure 3).

### CRH increases pneumolysin (Ply) mRNA gene expression

**Figure 4:**
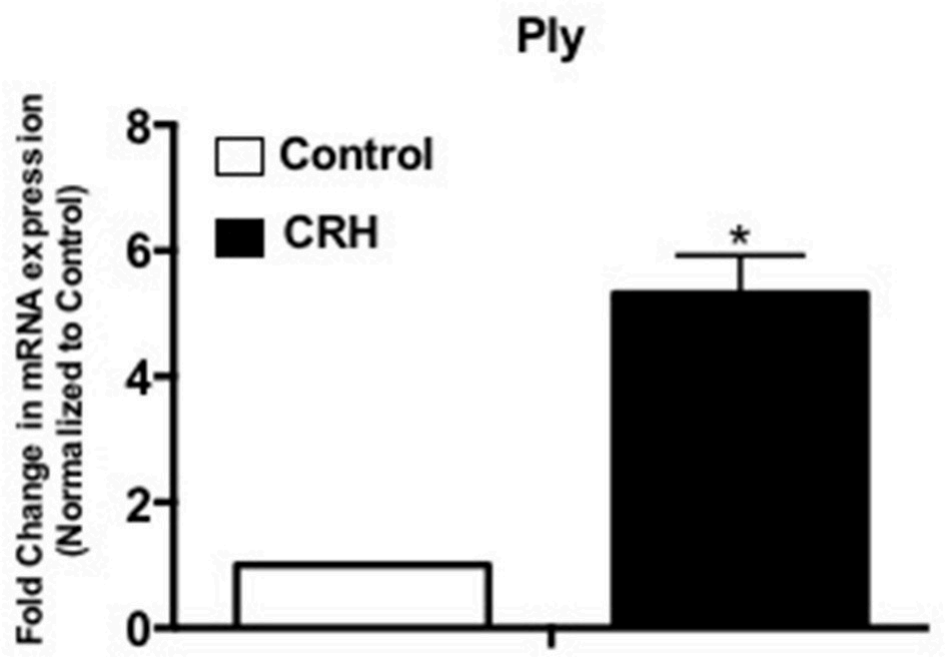
CRH increases Ply mRNA expression of Serotype 1 pneumococcal strain. Serotype 1 pneumococcal strain was grown in the presence or absence of CRH (4.0 × 10^−4^ mM/μl) for 5 h. Ply gene expression was determined by quantitative Real-Time PCR analysis. Data represents mean (*n* = 3) ± standard deviation in the fold increase in Ply of mRNA expression compared to control. Asterisks (^*^) indicate significant (*P* ≤ 0.05) difference between CRH and control.

As a non-membrane associated peptide, pneumolysin is prominently associated with disease pathogenesis of *S. pneumoniae*. Previous studies suggest that pneumolysin can influence immune and inflammatory responses contributing to sepsis and septic shock (Malley et al., 2003; Alhamdi et al., 2015). Our results show that CRH exposure significantly increases Ply mRNA gene expression at 3 h (Figure 4).

## Discussion

HPA-derived CRH mediates immune and inflammatory responses by control of cortisol (corticosterone in mice) release from the adrenals, which target glucocorticoid receptors expressed on peripheral tissues and immune cells. This causes a down-regulation of signaling pathways that suppress immune and inflammatory proteins and secreted factors (Silverman et al., 2005; Nezi et al., 2015). In addition, studies have shown that CRH can control immune and inflammatory responses directly through peripheral CRH expression and cognate CRH receptor ligation in peripheral tissues (Mastorakos et al., 2006; Nezi et al., 2015). Little is known however, regarding a bacterial species' response to CRH and other neuroendocrine factors as determinants of its virulence and pathogenicity. In previous studies, we were the first to demonstrate, to our knowledge, that CRH could directly impact *S. pneumoniae's* growth and *in vivo* pathogenesis (Ndjom and Jones, 2015). The results of the current study indicate that CRH potentiates morphological, genetic and functional properties vital to *S. pneumoniae's* virulence.

The transition from asymptomatic carriage to a pathogenic phenotype is dependent on a complex array of biochemical environmental cues that can influence the growth characteristics of *S. pneumoniae* (Cortes et al., 2015). Previous studies have demonstrated that changes in environments can modulate numerous proteins and carbohydrates that modify cell attachment, colonization and invasion that dictate biofilm formation and persistence in host (Allegrucci et al., 2006; Sanchez et al., 2011; Allan et al., 2014). In the current study, *in vitro* CRH exposure was found to accentuate growth when introduced during log phase. In contrast, CRH exposure during lag phase did not impact bacterial growth compared to control. We believe these results suggest the potential for CRH to modulate metabolic responses influential in the transition of growth status. In support, Raymond et al, elucidated by protein profiling, putative gene targets responsible for nascent phasic growth of *S. pneumoniae* observed during lag phase (Allan et al., 2014). Understanding however, that growth and biofilm formation involves a complex multistep process requires further study.

The current licensed 23-valent polysaccharide pneumococcal vaccine contains 23 purified capsular polysaccharide antigens of *S. pneumoniae* serotypes including serotype 1 used in this study (Jedrzejas, 2001). Proteins and enzymes embedded on the surface of *S. pneumoniae* have been shown to significantly contribute to its pathogenesis involving direct contact with host tissues, biofilm formation, and in concealing bacterial-associated molecules from host defense mechanisms (AlonsoDeVelasco et al., 1995; Mitchell and Mitchell, 2010). We found that CRH induced a significant increase in capsule thickness, indicating that this neuropeptide could potentially impact pneumococcal morphological phenotypes. Similarly, serotypes 3, 19A, and 23F were also found to increase capsule size (Supplementary Figure 1). Environmental factors impact capsule phenotype that can dictate pneumococcal transition from carriage within the nasal passages to a more motile invasive phenotype (Selinger et al., 1979; Kim and Weiser, 1998; Weiser et al., 2001; Hammerschmidt et al., 2005). Previous studies have characterized thinner capsular phenotypes to be associated with early colonization. In fact, the more sessile phenotypes resemble a thicker capsule present during invasive disease (Kim and Weiser, 1998; Kim et al., 1999). Furthermore, Lee et al. (1991) and Weinberger et al. (2009) reported that increased capsular thickness was advantageous to *S. pneumoniae*, as it allows the bacteria to evade phagocytosis by immune cells and thus, increase its carriage within the host environment. While this is the first study to examine capsule formation in response to CRH, previous studies have demonstrated catecholamine-effects on bacterial biofilm formation. Specifically, Sandrini et al. showed that norepinephrine (NE) exposure had no effect on capsule formation compared to bacterial growth, but increased pneumococcal attachment (Sandrini et al., 2014). In addition, Marks et al., found that NE induced the release of bacteria from biofilms (Marks et al., 2013). Our finding that CRH increased capsule growth is consistent with potentiating the invasive phenotype of serotype 1. In that other serotypes tested responded similarly to CRH in terms of capsular formation suggest CRH could be a global regulator of capsular structure and therefore relevant to pneumococcal pathogenicity. The capsular polysaccharide of *S. pneumoniae* mainly consists of repeating oligosaccharide units composed of two to eight monosaccharides linked by glycosidic bonds in which capsular diversity is generated (van Selm et al., 2003). To date, the genes regulating capsular synthesis for most serotypes including serotype 1 have been sequenced (van Selm et al., 2003). Such information will benefit how CRH may influence the genetic regulatory networks encoding the capsule biosynthesis pathways.

Although antimicrobial agents such as penicillin have been proven effective in reducing the risk from pneumococcal disease, resistant strains continue to increase in the United States and worldwide (Hackel et al., 2013; Musher, 2016). Studies have documented in some areas as much as 35% of pneumococcal isolates are resistant to penicillin (Arnold et al., 1996; Schrag et al., 2004; Low, 2005). We therefore determined the potential influence of CRH on *S. pneumoniae's* sensitivity to penicillin/streptomycin. Serotype 1 exposed to CRH resulted in greater than two-fold resistance to penicillin/streptomycin defined by the minimal inhibitory concentration. In support, a previous study by Severin et al., reported that genetic elements related to penicillin binding-proteins may produce a reduction in penicillin affinity by genetic transformation (Severin et al., 1996). It is worth noting that all tested serotypes revealed the highest percent increase in capsular thickness compared to non-CRH exposed serotypes (Supplementary Figure 1). Thus, one might expect that CRH plays a role in polysaccharide structure by increased polymerization of repeating oligosaccharide units. Furthermore, an increased high molecular capsular mass structure could provide added protection against penicillin targets. This and other potential mechanisms of antibiotic resistance dictated by CRH and other neuroendocrine factors may prove significant given the ever-increasing antibiotic resistance among invasive pneumococcal serotypes.

The discovery of pneumolysin (Ply) and other pneumococcal peptides, has led to an insurgence of novel virulence factors useful in vaccine development (Briles et al., 1997; Jedrzejas, 2001; Lin et al., 2015). Ply is a non-surface bound 53-kDA protein that penetrates the physical defense of the host tissue barriers (Hirst et al., 2004). Ply is a cytotoxic enzyme which mainly disrupts host ciliary function and tight junctions of bronchial epithelia that impairs mucus clearance in the lower respiratory airways (Rayner et al., 1995). We found CRH to increase Ply mRNA expression, indicating a non-membrane associated response by *S. pneumoniae* to CRH. In addition, Ply mRNA expression varied among serotypes 3, 19A, and 23F. Whereas serotypes 3 and 19A Ply expression was decreased in response to CRH, 23F Ply mRNA expression was significantly increased (Supplementary Figure 2). Ply is released in response to activation of surface autolysins. These enzymes degrade the peptidoglycan backbone of pneumococcus and are believed to be involved in cell growth turnover (Rogers et al., 1980). Interestingly, while CRH promoted capsular size, the increase in Ply gene expression suggest an inverse relationship between the activation of autolysins that would negatively influence cell wall growth; thus, contradicting the observed increase in Ply mRNA expression. Nevertheless, the induction of Ply in response to CRH suggest a mechanism through which pneumococcus may sense stress that elicits the induction of virulence gene expression of non-membrane bound proteins. Future studies that determine the temporal as well as serotype specificity of CRH-induced Ply protein secretion in relation to capsule and autolysin expression would confirm such direct or dichotomous relationships. Moreover, an in-depth study of the effects of CRH on the large class of non-membrane virulence factors will be important to fully understand its role in the pathophysiology of *S. pneumoniae*.

Much of the reports pertaining to neuroendocrine factors' role in *S. pneumoniae* virulence and pathogenicity have focused on catecholaminergic responses (Won and Ross, 1975; Gonzales et al., 2013; Sandrini et al., 2014). Yet, little is known regarding how glucocorticoids directly impact bacterial pathogenicity (Christ-Crain et al., 2007; Verbrugghe et al., 2011; Simard et al., 2014; Declercq et al., 2016). We have previously demonstrated a correlation between CRH exposure and increased disease severity *in vivo*. In this study, we begin to delineate direct mechanisms responsible for CRH's effect *on* key functional hallmarks of pneumococcal virulence. *S. pneumoniae* causes a variety of diseases among which, sepsis and septic shock remain a major health concern in the U.S and worldwide. Adequate treatment is becoming more complicated due to the emergence of increased resistance to commonly used antibiotics (Charpentier and Tuomanen, 2000). In addition, control of immunopathologies caused by aberrant immune and inflammatory responses remains a major mortality risk. Due to the current controversy surrounding the use of glucocorticoid administration, an in depth understanding of the implications of bacterial species' response to CRH and other glucocorticoids is warranted. We anticipate that defining pathways through which CRH regulates *S. pneumoniae's* physiology could reveal putative therapeutic targets as novel and alternative approaches to control adverse inflammatory responses. An increased knowledge of CRH interaction with bacterial pathogens in the present of antimicrobial agents will benefit the potential for novel antibiotics. In addition, information regarding CRH's role in mediation of membrane and non-membrane bound peptides could reveal novel target as vaccine candidates. Furthermore, studies defining the mechanisms by which *S. pneumoniae* recognizes and responds to CRH will help advance the field of bacterio-endocrinology. Resolving these points will have a broader impact in the prevention and treatment of infectious disease.

## Author contributions

HJ: as corresponding author directed and reviewed the design, execution, and data interpretation of all studies and affirmed the final draft of this paper for submission. CN: as lead author, developed and conducted, interpreted, and produced in its current version all of the studies within this paper and produced the final written version of this paper with advise from her co-authors. LK: as co-author assisted CN in the development and execution of the gene expression study and contributed to the interpretation of data findings relevant to the study.

### Conflict of interest statement

The authors declare that the research was conducted in the absence of any commercial or financial relationships that could be construed as a potential conflict of interest.

## References

[B1] Alhamdi Y. Neill D. R. Abrams S. T. Malak H. A. Yahya R. Barrett-Jolley R.. (2015). Circulating pneumolysin is a potent inducer of cardiac injury during pneumococcal infection. PLoS Pathog. 11:e1004836. 10.1371/journal.ppat.1004836 25973949 PMC4431880

[B2] Allan R. N. Skipp P. Jefferies J. Clarke S. C. Faust S. N. Hall-Stoodley L.. (2014). Pronounced metabolic changes in adaptation to biofilm growth by *Streptococcus pneumoniae*. PLoS ONE 9:e107015. 10.1371/journal.pone.0107015 25188255 PMC4154835

[B3] Allegrucci M. Hu F. Z. Shen K. Hayes J. Ehrlich G. D. Post J. C.. (2006). Phenotypic characterization of *Streptococcus pneumoniae* biofilm development. J. Bacteriol. 188, 2325–2335. 10.1128/JB.188.7.2325-2335.2006 16547018 PMC1428403

[B4] AlonsoDeVelasco E. Verheul A. F. Verhoef J. Snippe H. (1995). *Streptococcus pneumoniae*: virulence factors, pathogenesis, and vaccines. Microbiol. Rev. 59, 591–603. 8531887 10.1128/mr.59.4.591-603.1995 PMC239389

[B5] Annane D. (2011). Corticosteroids for severe sepsis: an evidence-based guide for physicians. Ann. Intensive Care 1:7. 10.1186/2110-5820-1-7 21906332 PMC3224490

[B6] Annane D. (2016). The role of ACTH and corticosteroids for sepsis and septic shock: an update. Front. Endocrinol. 7:70. 10.3389/fendo.2016.00070 27379022 PMC4913096

[B7] Annane D. Bellissant E. Bollaert P. E. Briegel J. Confalonieri M. De Gaudio R.. (2009). Corticosteroids in the treatment of severe sepsis and septic shock in adults: a systematic review. JAMA 301, 2362–2375. 10.1001/jama.2009.815 19509383

[B8] Annane D. Umberto Meduri G. Marik P. (2008). Critical illness-related corticosteroid insufficiency and community-acquired pneumonia: back to the future! Eur. Respir. J. 31, 1150–1152. 10.1183/09031936.00040908 18515553

[B9] Arnold K. E. Leggiadro R. J. Breiman R. F. Lipman H. B. Schwartz B. Appleton M. A.. (1996). Risk factors for carriage of drug-resistant *Streptococcus pneumoniae* among children in Memphis, Tennessee. J. Pediatr. 128, 757–764. 10.1016/S0022-3476(96)70326-8 8648533

[B10] Benou C. Wang Y. Imitola J. VanVlerken L. Chandras C. Karalis K. P.. (2005). Corticotropin-releasing hormone contributes to the peripheral inflammatory response in experimental autoimmune encephalomyelitis. J. Immunol. 174, 5407–5413. 10.4049/jimmunol.174.9.5407 15843539

[B11] Beutz M. A. Abraham E. (2005). Community-acquired pneumonia and sepsis. Clin. Chest Med. 26, 19–28. 10.1016/j.ccm.2004.10.015 15802162

[B12] Boomer J. S. To K. Chang K. C. Takasu O. Osborne D. F. Walton A. H.. (2011). Immunosuppression in patients who die of sepsis and multiple organ failure. JAMA 306, 2594–2605. 10.1001/jama.2011.1829 22187279 PMC3361243

[B13] Bosmann M. Ward P. A. (2013). The inflammatory response in sepsis. Trends Immunol. 34, 129–136. 10.1016/j.it.2012.09.004 23036432 PMC3543471

[B14] Briles D. E. Hollingshead S. K. Swiatlo E. Brooks-Walter A. Szalai A. Virolainen A.. (1997). PspA and PspC: their potential for use as pneumococcal vaccines. Microb. Drug Resist. 3, 401–408. 10.1089/mdr.1997.3.401 9442494

[B15] Butts C. L. Sternberg E. M. (2008). Neuroendocrine factors alter host defense by modulating immune function. Cell. Immunol. 252, 7–15. 10.1016/j.cellimm.2007.09.009 18329009 PMC2590632

[B16] Charpentier E. Tuomanen E. (2000). Mechanisms of antibiotic resistance and tolerance in *Streptococcus pneumoniae*. Microbes Infect. 2, 1855–1864. 10.1016/S1286-4579(00)01345-9 11165930

[B17] Chen X.-H. Yin Y.-J. Zhang J.-X. (2011). Sepsis and immune response. World J. Emer. Med. 2, 88–92. 10.5847/wjem.j.1920-8642.2011.02.002 25214990 PMC4129694

[B18] Christ-Crain M. Stolz D. Jutla S. Couppis O. Muller C. Bingisser R.. (2007). Free and total cortisol levels as predictors of severity and outcome in community-acquired pneumonia. Am. J. Respir. Crit. Care Med. 176, 913–920. 10.1164/rccm.200702-307OC 17702966

[B19] Cope E. K. Goldstein-Daruech N. Kofonow J. M. Christensen L. McDermott B. Monroy F.. (2011). Regulation of virulence gene expression resulting from *Streptococcus pneumoniae* and nontypeable Haemophilus influenzae interactions in chronic disease. PLoS ONE 6:e28523. 10.1371/journal.pone.0028523 22162775 PMC3230614

[B20] Cortes P. R. Piñas G. E. Cian M. B. Yandar N. Echenique J. (2015). Stress-triggered signaling affecting survival or suicide of Streptococcus pneumoniae. Int. J. Med. Microbiol. 305, 157–169. 10.1016/j.ijmm.2014.12.002 25543170

[B21] Crofford L. J. Sano H. Karalis K. Webster E. L. Goldmuntz E. A. Chrousos G. P.. (1992). Local secretion of corticotropin-releasing hormone in the joints of Lewis rats with inflammatory arthritis. J. Clin. Invest. 90, 2555–2564. 10.1172/JCI116150 1281840 PMC443415

[B22] Declercq A. M. Aerts J. Ampe B. Haesebrouck F. De Saeger S. Decostere A. (2016). Cortisol directly impacts Flavobacterium columnare *in vitro* growth characteristics. Vet. Res. 47, 84. 10.1186/s13567-016-0370-9 27530746 PMC4987970

[B23] den Brinker M. Joosten K. F. Liem O. de Jong F. H. Hop W. C. Hazelzet J. A.. (2005). Adrenal insufficiency in meningococcal sepsis: bioavailable cortisol levels and impact of interleukin-6 levels and intubation with etomidate on adrenal function and mortality. J. Clin. Endocrinol. Metab. 90, 5110–5117. 10.1210/jc.2005-1107 15985474

[B24] Gonzales X. F. Castillo-Rojas G. Castillo-Rodal A. I. Tuomanen E. Lopez-Vidal Y. (2013). Catecholamine norepinephrine diminishes lung epithelial cell adhesion of *Streptococcus pneumoniae* by binding iron. Microbiology 159(Pt 11), 2333–2341. 10.1099/mic.0.065607-0 23963302

[B25] Gonzales X. F. Deshmukh A. Pulse M. Johnson K. Jones H. P. (2008). Stress-induced differences in primary and secondary resistance against bacterial sepsis corresponds with diverse corticotropin releasing hormone receptor expression by pulmonary CD11c+ MHC II+ and CD11c- MHC II+ APCs. Brain Behav. Immun. 22, 552–564. 10.1016/j.bbi.2007.11.005 18166336 PMC2849292

[B26] Hackel M. Lascols C. Bouchillon S. Hilton B. Morgenstern D. Purdy J. (2013). Serotype prevalence and antibiotic resistance in *Streptococcus pneumoniae* clinical isolates among global populations. Vaccine 31, 4881–4887. 10.1016/j.vaccine.2013.07.054 23928466

[B27] Hall M. J. Williams S. N. DeFrances C. J. Golosinskiy A. (2011). Inpatient care for septicemia or sepsis: a challenge for patients and hospitals. NCHS Data Brief. Available online at: https://www.cdc.gov/nchs/data/databriefs/db62.htm 22142805

[B28] Hammerschmidt S. Wolff S. Hocke A. Rosseau S. Müller E. Rohde M. (2005). Illustration of pneumococcal polysaccharide capsule during adherence and invasion of epithelial cells. Infect. Immun. 73, 4653–4667. 10.1128/IAI.73.8.4653-4667.2005 16040978 PMC1201225

[B29] Hifumi T. Fujishima S. Abe T. Kiriu N. Inoue J. Kato H.. (2016). Prognostic factors of *Streptococcus pneumoniae* infection in adults. Am. J. Emerg. Med. 34, 202–206. 10.1016/j.ajem.2015.10.025 26508390

[B30] Hirst R. A. Kadioglu A. O'Callaghan C. Andrew P. W. (2004). The role of pneumolysin in pneumococcal pneumonia and meningitis. Clin. Exp. Immunol. 138, 195–201. 10.1111/j.1365-2249.2004.02611.x 15498026 PMC1809205

[B31] Hughes R. B. Smith A. C. (2007). Capsule Stain Protocols [Online]. ASM MicrobeLibrary. Available online at: http://www.asmscience.org/content/education/protocol/protocol.3041

[B32] Jedrzejas M. J. (2001). Pneumococcal virulence factors: structure and function. Microbiol. Mol. Biol. Rev. 65, 187–207. 10.1128/MMBR.65.2.187-207.2001 11381099 PMC99024

[B33] Kalantaridou S. Makrigiannakis A. Zoumakis E. Chrousos G. P. (2007). Peripheral corticotropin-releasing hormone is produced in the immune and reproductive systems: actions, potential roles and clinical implications. Front. Biosci. 12, 572–580. 10.2741/2083 17127318

[B34] Kim B. J. Kayembe K. Simecka J. W. Pulse M. Jones H. P. (2011). Corticotropin-releasing hormone receptor-1 and 2 activity produces divergent resistance against stress-induced pulmonary *Streptococcus pneumoniae* infection. J. Neuroimmunol. 237, 57–65. 10.1016/j.jneuroim.2011.06.016 21774994 PMC5715473

[B35] Kim J. O. Weiser J. N. (1998). Association of intrastrain phase variation in quantity of capsular polysaccharide and teichoic acid with the virulence of *Streptococcus pneumoniae*. J. Infect. Dis. 177, 368–377. 10.1086/514205 9466523

[B36] Kim J. O. Romero-Steiner S. Sørensen U. B. S. Blom J. Carvalho M. Barnard S.. (1999). Relationship between cell surface carbohydrates and intrastrain variation on opsonophagocytosis of *Streptococcus pneumoniae*. Infect. Immun. 67, 2327–2333. 10225891 10.1128/iai.67.5.2327-2333.1999 PMC115974

[B37] Kokkotou E. Torres D. Moss A. C. O'Brien M. Grigoriadis D. E. Karalis K.. (2006). Corticotropin-releasing hormone receptor 2-deficient mice have reduced intestinal inflammatory responses. J. Immunol. 177, 3355–3361. 10.4049/jimmunol.177.5.3355 16920976

[B38] Lee C. J. Banks S. D. Li J. P. (1991). Virulence, immunity, and vaccine related to *Streptococcus pneumoniae*. Crit. Rev. Microbiol. 18, 89–114. 10.3109/10408419109113510 1930677

[B39] Lin H. Peng Y. Lin Z. Zhang S. Guo Y. (2015). Development of a conjugate vaccine against invasive pneumococcal disease based on capsular polysaccharides coupled with PspA/family 1 protein of *Streptococcus pneumoniae*. Microb. Pathog. 83–84, 35–40. 10.1016/j.micpath.2015.04.006 25959527

[B40] Low D. E. (2005). Changing trends in antimicrobial-resistant pneumococci: it's not all bad news. Clin. Infect. Dis. 41(Suppl. 4), S228–S233. 10.1086/430782 16032557

[B41] Lynch J. P. Martinez F. J. (2002). Clinical relevance of macrolide-resistant *Streptococcus pneumoniae* for community-acquired pneumonia. Clin. Infect. Dis. 34(Suppl. 1), S27–S46. 10.1086/324527 11810608

[B42] Malley R. Henneke P. Morse S. C. Cieslewicz M. J. Lipsitch M. Thompson C. M.. (2003). Recognition of pneumolysin by Toll-like receptor 4 confers resistance to pneumococcal infection. Proc. Natl. Acad. Sci. U.S.A. 100, 1966–1971. 10.1073/pnas.0435928100 12569171 PMC149942

[B43] Marik P. E. Zaloga G. P. (2002). Adrenal insufficiency in the critically ill: a new look at an old problem. Chest 122, 1784–1796. 10.1378/chest.122.5.1784 12426284

[B44] Marks L. R. Davidson B. A. Knight P. R. Hakansson A. P. (2013). Interkingdom signaling induces *Streptococcus pneumoniae* biofilm dispersion and transition from asymptomatic colonization to disease. mBio 4:e00438-13. 10.1128/mBio.00438-13 23882016 PMC3735180

[B45] Martin G. S. (2012). Sepsis, severe sepsis and septic shock: changes in incidence, pathogens and outcomes. Expert Rev. Anti Infect. Ther. 10, 701–706. 10.1586/eri.12.50 22734959 PMC3488423

[B46] Mastorakos G. Karoutsou E. I. Mizamtsidi M. (2006). Corticotropin releasing hormone and the immune/inflammatory response. Eur. J. Endocrinol. 155(Suppl. 1), S77–S84. 10.1530/eje.1.02243

[B47] Minneci P. C. Deans K. J. Eichacker P. Q. Natanson C. (2009). The effects of steroids during sepsis depend on dose and severity of illness: an updated meta-analysis. Clin. Microbiol. Infect. 15, 308–318. 10.1111/j.1469-0691.2009.02752.x 19416302 PMC3383780

[B48] Mitchell A. M. Mitchell T. J. (2010). *Streptococcus pneumoniae*: virulence factors and variation. Clin. Microbiol. Infect. 16, 411–418. 10.1111/j.1469-0691.2010.03183.x 20132250

[B49] Moran J. L. Graham P. L. Rockliff S. Bersten A. D. (2010). Updating the evidence for the role of corticosteroids in severe sepsis and septic shock: a Bayesian meta-analytic perspective. Crit. Care 14:R134. 10.1186/cc9182 20626892 PMC2945102

[B50] Musher D. M. (2016). Resistance of Streptococcus pneumoniae to Beta-Lactam Antibiotics, ed Sexton D. J. UpToDate. Available online at: https://www.uptodate.com/contents/resistance-of-streptococcus-pneumoniae-to-beta-lactam-antibiotics

[B51] Nasa P. Juneja D. Singh O. (2012). Severe sepsis and septic shock in the elderly: an overview. World J. Crit. Care Med. 1, 23–30. 10.5492/wjccm.v1.i1.23 24701398 PMC3956061

[B52] Ndjom C. G. Jones H. P. (2015). CRH promotes *S. pneumoniae* growth in vitro and increases lung carriage in mice. Front. Microbiol. 6:279. 10.3389/fmicb.2015.00279 25904910 PMC4389549

[B53] Nezi M. Mastorakos G. Mouslech Z. (2015). Corticotropin releasing hormone and the immune/inflammatory response, in Endotext [Internet], eds De Groot L. J. Chrousos G. Dungan K. (South Dartmouth: MDText.com, Inc.).

[B54] Page D. B. Donnelly J. P. Wang H. E. (2015). Community-, healthcare-, and hospital-acquired severe sepsis hospitalizations in the university healthsystem consortium. Crit. Care Med. 43, 1945–1951. 10.1097/CCM.0000000000001164 26110490 PMC4537676

[B55] Prigent H. Maxime V. Annane D. (2004). Clinical review: corticotherapy in sepsis. Crit. Care 8, 122–129. 10.1186/cc2374 15025773 PMC420022

[B56] Procaccini C. Pucino V. De Rosa V. Marone G. Matarese G. (2014). Neuro-endocrine networks controlling immune system in health and disease. Front. Immunol. 5:143. 10.3389/fimmu.2014.00143 24778633 PMC3985001

[B57] Rappleye E. (2015). Average Cost Per Inpatient Day Across 50 States [Online]. Beckker's Hospital Review. Available online at: http://www.beckershospitalreview.com/finance/average-cost-per-inpatient-day-across-50-states.html

[B58] Rayner C. F. Jackson A. D. Rutman A. Dewar A. Mitchell T. J. Andrew P. W.. (1995). Interaction of pneumolysin-sufficient and -deficient isogenic variants of *Streptococcus pneumoniae* with human respiratory mucosa. Infect. Immun. 63, 442–447. 7822008 10.1128/iai.63.2.442-447.1995 PMC173015

[B59] Riyaz M. Manuel R. Joseph N K. (2014). Importance of serum procalcitonin in febrile neutropenia. J. Evol. Med. Dental Sci. 3, 8012–8018. 10.14260/jemds/2014/3004

[B60] Rogers P. D. Liu T. T. Barker K. S. Hilliard G. M. English B. K. Thornton J.. (2007). Gene expression profiling of the response of *Streptococcus pneumoniae* to penicillin. J. Antimicrob. Chemother. 59, 616–626. 10.1093/jac/dkl560 17339278

[B61] Rogers H. J. Perkins H. R. Ward J. B. (1980). Formation of cell wall polymers, in Microbial Cell Wall and Membranes, ed Nombela C. (London: Chapman & Hall), 437–460.

[B62] Russell J. A. (2006). Management of sepsis. New Engl. J. Med. 355, 1699–1713. 10.1056/NEJMra043632 17050894

[B63] Sam S. Corbridge T. C. Mokhlesi B. Comellas A. P. Molitch M. E. (2004). Cortisol levels and mortality in severe sepsis. Clin. Endocrinol. 60, 29–35. 10.1111/j.1365-2265.2004.01923.x 14678284

[B64] Sanchez C. J. Kumar N. Lizcano A. Shivshankar P. Dunning Hotopp J. C. Jorgensen J. H.. (2011). *Streptococcus pneumoniae* in biofilms are unable to cause invasive disease due to altered virulence determinant production. PLoS ONE 6:e28738. 10.1371/journal.pone.0028738 22174882 PMC3234282

[B65] Sandrini S. Alghofaili F. Freestone P. Yesilkaya H. (2014). Host stress hormone norepinephrine stimulates pneumococcal growth, biofilm formation and virulence gene expression. BMC Microbiol. 14:180. 10.1186/1471-2180-14-180 24996423 PMC4105557

[B66] Schrag S. J. McGee L. Whitney C. G. Beall B. Craig A. S. Choate M. E.. (2004). Emergence of *Streptococcus pneumoniae* with very-high-level resistance to penicillin. Antimicrob. Agents Chemother. 48, 3016–3023. 10.1128/AAC.48.8.3016-3023.2004 15273115 PMC478489

[B67] Selinger D. S. Selinger R. C. Reed W. P. (1979). Resistance to infection of the external eye: the role of tears. Surv. Ophthalmol. 24, 33–38. 10.1016/0039-6257(79)90145-0 384573

[B68] Severin A. Figueiredo A. M. Tomasz A. (1996). Separation of abnormal cell wall composition from penicillin resistance through genetic transformation of *Streptococcus pneumoniae*. J. Bacteriol. 178, 1788–1792. 10.1128/jb.178.7.1788-1792.1996 8606149 PMC177870

[B69] Silverman M. N. Pearce B. D. Biron C. A. Miller A. H. (2005). Immune modulation of the hypothalamic-pituitary-adrenal (HPA) axis during viral infection. Viral Immunol. 18, 41–78. 10.1089/vim.2005.18.41 15802953 PMC1224723

[B70] Simard M. Hill L. A. Underhill C. M. Keller B. O. Villanueva I. Hancock R. E.. (2014). Pseudomonas aeruginosa elastase disrupts the cortisol-binding activity of corticosteroid-binding globulin. Endocrinology 155, 2900–2908. 10.1210/en.2014-1055 24848868 PMC4098004

[B71] Sligl W. I. Milner D. A. Jr. Sundar S. Mphatswe W. Majumdar S. R. (2009). Safety and efficacy of corticosteroids for the treatment of septic shock: a systematic review and meta-analysis. Clin. Infect. Dis. 49, 93–101. 10.1086/599343 19489712

[B72] ThyagaRajan S. Priyanka H. P. (2012). Bidirectional communication between the neuroendocrine system and the immune system: relevance to health and diseases. Ann. Neurosci. 19, 40–46. 10.5214/ans.0972.7531.180410 25205962 PMC4117073

[B73] van Selm S. van Cann L. M. Kolkman M. A. B. van der Zeijst B. A. M. van Putten J. P. M. (2003). Genetic basis for the structural difference between *Streptococcus pneumoniae* serotype 15B and 15C capsular polysaccharides. Infect. Immun. 71, 6192–6198. 10.1128/IAI.71.11.6192-6198.2003 14573636 PMC219561

[B74] van Tol E. A. Petrusz P. Lund P. K. Yamauchi M. Sartor R. B. (1996). Local production of corticotropin releasing hormone is increased in experimental intestinal inflammation in rats. Gut 39, 385–392. 10.1136/gut.39.3.385 8949642 PMC1383344

[B75] Verbrugghe E. Boyen F. Van Parys A. Van Deun K. Croubels S. Thompson A.. (2011). Stress induced Salmonella Typhimurium recrudescence in pigs coincides with cortisol induced increased intracellular proliferation in macrophages. Vet. Res. 42:118. 10.1186/1297-9716-42-118 22151081 PMC3256119

[B76] Volbeda M. Wetterslev J. Gluud C. Zijlstra J. G. van der Horst I. C. Keus F. (2015). Glucocorticosteroids for sepsis: systematic review with meta-analysis and trial sequential analysis. Intensive Care Med. 41, 1220–1234. 10.1007/s00134-015-3899-6 26100123 PMC4483251

[B77] Weinberger D. M. Trzcinski K. Lu Y. J. Bogaert D. Brandes A. Galagan J.. (2009). Pneumococcal capsular polysaccharide structure predicts serotype prevalence. PLoS Pathog. 5:e1000476. 10.1371/journal.ppat.1000476 19521509 PMC2689349

[B78] Weiser J. N. Bae D. Epino H. Gordon S. B. Kapoor M. Zenewicz L. A.. (2001). Changes in availability of oxygen accentuate differences in capsular polysaccharide expression by phenotypic variants and clinical isolates of *Streptococcus pneumoniae*. Infect. Immun. 69, 5430–5439. 10.1128/IAI.69.9.5430-5439.2001 11500414 PMC98654

[B79] Wilson J. W. Schurr M. J. LeBlanc C. L. Ramamurthy R. Buchanan K. L. Nickerson C. A. (2002). Mechanisms of bacterial pathogenicity. Postgrad. Med. J. 78, 216–224. 10.1136/pmj.78.918.216 11930024 PMC1742320

[B80] Wilson M. Seymour R. Henderson B. (1998). Bacterial perturbation of cytokine networks. Infect. Immun. 66, 2401–2409. 9596695 10.1128/iai.66.6.2401-2409.1998 PMC108217

[B81] Won W. D. Ross H. C. (1975). Catecholamine and phagocytic responses in infected mice exposed to hyperbaric helium-oxygen atmospheres. Aviat. Space Environ. Med. 46, 191–193. 1115719

[B82] Wood K. A. Angus D. C. (2004). Pharmacoeconomic implications of new therapies in sepsis. Pharmacoeconomics 22, 895–906. 10.2165/00019053-200422140-00001 15362927

