# Supplementary material for: CRH Affects the Phenotypic Expression of Sepsis-Associated Virulence Factors by Streptococcus pneumoniae Serotype 1 In vitro
Source: Front Cell Infect Microbiol. 2017 Jun 22;7:263. doi: 10.3389/fcimb.2017.00263 (PMC5479890; doi:10.3389/fcimb.2017.00263)
Supplement: Supplementary file 1 [file DataSheet1.docx]

Supplementary Material

***Streptococcus pneumoniae* virulence factors are influenced by Corticotropin releasing hormone as potential determinants of sepsis and septic shock**

Colette Georgette Ngo Ndjom^1^, Lindsay Vermont Kantor^2^, Harlan Pierre Jones^1*^

*** Correspondence:** Harlan P. Jones: [harlan.jones@unthsc.edu](mailto:harlan.jones@unthsc.edu)

# Supplementary Figures


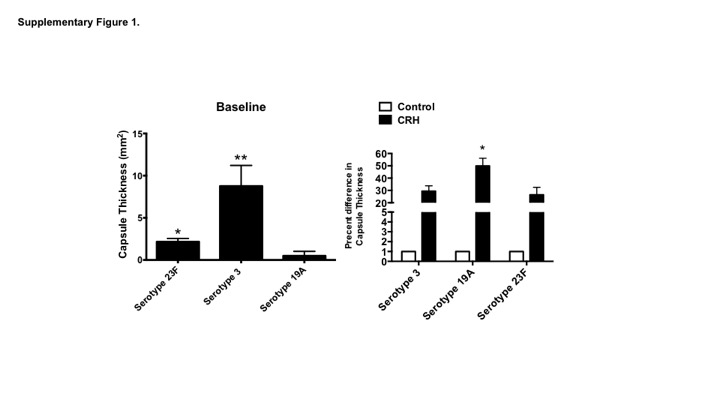


**Supplementary Figure 1. Comparative capsule thickness of serotypes 3, 19A and 23F in response to CRH.** 10^8^ organisms of pneumococcal serotypes 3, 19A and 23F was grown in the presence or absence of CRH (4.0 x 10^-4^ mM/µl) for 5 hours. Baseline Capsular thickness (left) and capsular thickness in response to CRH (right) was determined by calculating the absolute difference between total diameter of outer and inner capsular membranes. Bars represent the mean (n=9) ± standard deviation in the percent difference in capsular diameters of CRH-treated organisms compared to control. Asterisks (^∗^) indicate significant (*P* ≤ 0.05) differences between control and (**) indicate significant (*P* ≤ 0.05) between all experimental groups.

**
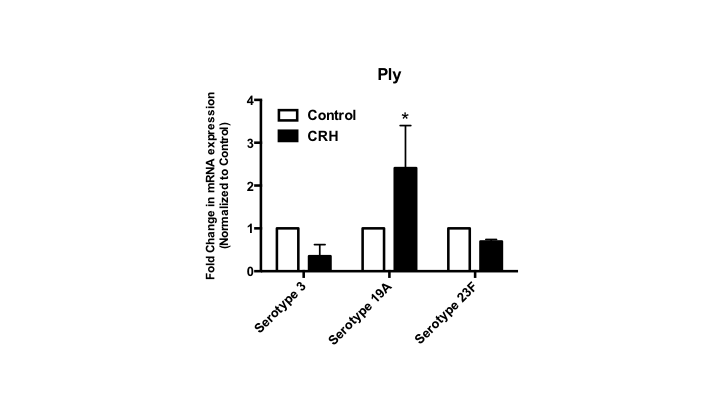
**

**Supplementary Figure 2. CRH does not increase pneumolysin (ply) expression.** Pneumococcal serotypes 3, 19A and 23F (1 X 10^6^ organisms) were grown in the presence or absence of CRH (4.0 x 10^-4^ mM/µl) for 5 hours. Ply gene expression was determined by quantitative Real-Time PCR analysis. Data represents mean (n=3) ± standard deviation in the fold increase in Ply2 of mRNA expression compared to control. Asterisks (^∗^) indicate significant (*P* ≤ 0.05) difference between CRH and control.
